# HECTD1 controls the protein level of IQGAP1 to regulate the dynamics of adhesive structures

**DOI:** 10.1186/s12964-016-0156-8

**Published:** 2017-01-05

**Authors:** Xiaoli Shen, Zanhui Jia, Donato D’Alonzo, Xinggang Wang, Elisabeth Bruder, Fabienne Hélène Emch, Christian De Geyter, Hong Zhang

**Affiliations:** 1 Department of Biomedicine, University Hospital, University of Basel, Basel, Switzerland; 2 Pathologie, Universitätsspital Basel, Schönbeinstrasse 40, CH-4031 Basel, Switzerland; 3 Clinic of Gynecological Endocrinology and Reproductive Medicine, University Hospital, University of Basel, Basel, Switzerland; 4 Department of Biomedicine, University of Basel, Hebelstra. 20, CH-4031 Basel, Switzerland; 5 Present Address: Chongqing Reproductive and Genetics Institute, 64 Jing Tang ST, Yu Zhong District Chongqing, 400013 China; 6 Present Address: 2nd hospital of Jilin University, Changchun, China

**Keywords:** HECTD1, Cell movement, Cell spreading, Focal adhesions, IQGAP1, Ubiquitination

## Abstract

**Background:**

Cell migration including collective cell movement and individual cell migration are crucial factors in embryogenesis. During the spreading/migration of cells, several types of adhesive structures physically interacting with the extracellular matrix (ECM) or with another cell have been described and the formation and maturation of adhesion structures are coordinated, however the molecular pathways involved are still not fully understood.

**Results:**

We generated a mouse embryonic fibroblast line (MEF) from homozygous mutant (*Hectd1* ^*R/R*^, *Hectd1* ^*Gt(RRC200)*^) mouse of the E3 ubiquitin ligase for inhibin B receptor (*Hectd1*). Detailed examination of cell motion on MEF cells demonstrated that loss of Hectd1 resulted in accelerated cell spreading and migration but impaired directionality of migration. In *Hectd1* ^*R/R*^ cells paxillin and zyxin were largely mis-localized, whereas their expression levels were unchanged. In addition the formation of focal adhesions (FAs) was impaired and the focal complexes (FXs) were increased. We further identified HECTD1 as a key regulator of IQGAP1. IQGAP1 co-localized together with HECTD1 in the leading edge of cells. HECTD1 interacted with IQGAP1 and regulated its degradation through ubiquitination. Over-expression of IQGAP1 in control MEF phenocopied the spreading and migration defects of *Hectd1* ^*R/R*^ cells. In contrast, siRNA-mediated knockdown of IQGAP1 rescued the defects in cellular movement of *Hectd1* ^*R/R*^ cells.

**Conclusions:**

The E3 ligase activity of Hectd1 regulates the protein level of IQGAP1 through ubiquitination and therefore mediates the dynamics of FXs including the recruitment of paxillin and actinin. IQGAP1 is one of the effectors of HECTD1.

**Electronic supplementary material:**

The online version of this article (doi:10.1186/s12964-016-0156-8) contains supplementary material, which is available to authorized users.

## Background

Cell migration including collective cell movement and individual cell migration are crucial factors in embryogenesis [1, 2], as best exemplified in neurulation [3, 4]. Generally, cell migration has been conceptualized as a cyclic process [5], in which a spreading phase is followed by migration involving actin polymerization and myosin contraction. Various mechanisms have been proposed for the regulation of cell spreading/migration, including active C-terminal Src kinase (CSK) remodeling [6], activation of focal adhesion kinase (FAK) and APR 2/3 [7], actin polymerization and the development of contractile forces [8, 9].

During the spreading/migration of cells in culture several types of adhesive structures physically interacting with the extracellular matrix (ECM) or with another cell have been described [10]. Owing to their highly dynamic nature and size, nascent adhesive structures and FXs typically are sized smaller than 1 μm^2^ [11]. As cells migrate, these structures either disappear or develop to mature FAs, which are large in size (>5 μm^2^). Although it is clear that the formation and maturation of adhesion structures are coordinated, the molecular pathways involved are still not fully understood [12].

EULIR was first identified as an E3 ubiquitin ligase for the putative inhibin B receptor in our laboratory [13], but international nomenclature later renamed EULIR to HECTD1. Sarkar and Zohn suggested that HSP90 is a binding partner of HECTD1 and that increased secretion of HSP90 in the cranial mesenchyme of HECTD1-mutants is in part responsible for the altered organization and behavior of these cells [14]. Tran and coworkers suggested that HECTD1 promotes the interaction of the adenomatous polyposis coli (APC) protein with Axin to negatively regulate Wnt signaling through Lys-63 polyubiquitination [15]. We found that knockdown of HECTD1 expression by siRNAs increased the migration velocity and membrane ruffling of HeLa cells. However during the course of our studies, Sarkar and Zohn demonstrated that *opm* mice increased the cranial mesenchyme cell migration [16, 17] but the findings from Li and coworkers showed that knockdown of HECTD1 inhibits the migration of breast cancer MDA-MB-231 cells [18]. To resolve this contradictory issue, we have used the Hectd1 homozygous mutant (*Hectd1* ^*R/R*^) mouse embryonic fibroblasts (MEF) generated from a gene-trap mouse embryonic stem (ES) cell line RRC200 (BayGenomics, San Francisco, CA, USA), for cell migration studies.

IQGAP1 belongs to the IQGAPs family of scaffold proteins. Despite the homology of amino-acid sequence with GAP, IQGAP1 does not exert any GTP hydrolysis activity [19–21]. In eukaryotic cells, IQGAP1 localizes to actin-containing structures such as lamellipodia, membrane ruffles and cell-to-cell adhesions. As such, IQGAP1 is involved in regulating cellular motility and morphogenesis [22]. Under normal conditions, through its coordinating with small GTPase, Rac1, RhoA and CDC42, IQGAP1 supports cell movement via regulating adherens junctions, actin filaments and microtubules. Initially, IQGAP1 was identified as a target of Rac1 and CDC42. In addition, activation of Rac and CDC42 in response to stimulation signals leads to the recruitment of IQGAP1, APC and CLIP-170, forming a complex which connects to the actin cytoskeleton and microtubules promoting cell polarization and directional cell migration [23–25]. Another mechanism proposed that IQGAP1 requires PIPKIγ for targeting to the leading edge of migrating cells and be activated specifically by PIP2 to promote actin polymerization and cell migration [26]. In contrast, IQGAP1 may also negatively impact on cell migration. One study demonstrated that IQGAP1 suppresses TβRII- and TGF-β-dependent myofibroblastic differentiation in tumors thereby inhibiting tumor growth [27]. Besides, anti-GTPase activity of IQGAP1 sustains the amount of GTP-bound Rac1 at sites of cell-to-cell contact, resulting in stable adhesion [28]. Recently, IQGAP1 was found to localize in FAs [29, 30] and in FXs together with integrin-linked kinase ILK [31]. Schiefermeier and coworkers reported that IQGAP1 interacts with FA proteins [32]. However, whether IQGAP1 is directly involved in regulation of the dynamics of FAs is still not known, neither is there anything known about its regulation. Through screening various ECMs and a number of adhesion proteins, we found that the stability of IQGAP1 is regulated by HECTD1.

We here propose a novel molecular mechanism explaining the role of Hectd1 in cell movement. Deficiency in Hectd1 results in failure to recruit phaxillin and zyxin to FAs thereby promoting rapid cell migration. Taking all data together, our results demonstrate that Hectd1 contributes to morphogenesis through the regulation of cell migration.

## Methods

### Aminals and mating scheme of mutant mouse

To generate *Hectd1* mutation mice [33], the gene-trap mouse embryonic stem (ES) cell line RRC200 on a 129 background (129P2/OlaHsd) obtained from (BayGenomics, San Francisco, CA, USA) was selected since the insertion site of the gene trap (β-geo) was mapped onto the intron 26 of the *Hectd1* gene, which includes the entire open reading frame but lacking the HECT1-domain (Additional file 1: Figure S1A). The ES cells were microinjected into blastocysts (C57BL/6NCrl × 6 J). Resulting agouti chimeric male mice were crossed with C57BL/6 female mice. Then F1 mice were intercrossed to generate more *Hectd1* ^*Gt(RRC200)Byg*^ mice for more than 10 generations.

### Generation and culture of mouse embryonic fibroblast (MEF) cells

On the day of E14.5, Hectd1 heterozygote mice were sacrificed. Then their embryos were photographed with a Leica M80 Stereomicroscope and plated on clean dishes. The trunks of the embryos were cut out with sterile scissors. The tissues were transferred to clean dishes and washed thoroughly with PBS, followed by gently mincing the tissues into small clumps of cells using two sterile needles. The cell clumps were digested with 500 μl Trypsin-EDTA at 37 °C for 20 min. After that, the digestion was stopped by 500 μl high glucose DMEM medium with 10% FBS, pipetted up and down for 5–10 times to disperse the clumps and centrifuged at 1000 rpm at room temperature for 1 min. Then the supernatant was removed through aspiration. The pellets were washed with PBS and repeated centrifuged. The pellets were dispersed by pipetting and grown on new culture plates in a humidified incubator at 37 °C, 5% CO2. MEF cells were sub-cultured when they reached 80–90% confluence.

### Cell culture and transfection

MEF cells were maintained in high glucose DMEM medium (HeLa cells in low glucose medium) with 10% FBS, 1% of Sodium Pyruvate, 1% of L-Glutaminate and 1% of Penicillin-Streptomycin. Cells were grown in a humidified incubator at 5% CO_2_ at 37 °C. MEF or HeLa cells used for transfection were pre-seeded 24 h in culture vessels. On the day of transfection, the confluence was 50–80%. Transfection of MEF or HeLa cells with plasmid DNA using Effectene reagent according to the protocol of Qiagen.

### Fibronectin coating

For cell spreading and migration assay, 24- well plates were coated with 2 μg/ml fibronectin (R&D, 1030-FN) in PBS overnight. For immunohistochemistry staining, glass coverslips were used for coating.

### Cell spreading assay

Cells were seeded on 6-well plates and incubated at 37 °C for 24 h before serum starvation overnight. Starved cells were counted and seeded on fibronectin pre-coated 24-well plates. The plate was immediately sent to time-lapse microscopy (Nikon IX81) pre-warmed to 37 °C and maintaining the CO_2_ level at 5%. Quickly adjusting the positions, the focus, the time interval and total time by CellSens software, the programme was initiated. Duration of spreading was analyzed from attachment to formation of leading protrusion. Cell spreading area was quantified by Image J software.

### Wound-healing assay

In monolayer wound-healing assays, 4 × 10^4^ cells were collected and plated in 24-well plate for 24 h. Cells were washed twice with PBS and continuously cultured for 24 h in growing medium containing 0.5% FBS, then cells were starved in serum free medium supplemented with 1 μM aphidicolin overnight. Then, cells were scratched with a 200 μl pipette tip, washed twice with PBS and placed into a complete medium containing 10% FBS and aphidicolin. The plate was immediately sent to time-lapse microscopy (Nikon IX81) pre-warmed to 37 °C and with 5% CO_2_. Migration images were taken at 10 min intervals for a period of 24 h with a 4× lens. Cell trajectories were measured by tracking the position of the cell over time using “Manual Tracking” plugin (Image J, v 2.0) and the cell velocity and straightness were determined by “Chemotaxis Tool” plugin (Image J, v 2.0). Cells that proliferate or that failed to migrate during the experimental period were not evaluated.

### Directionality of cell migration

The percentage of MTOC orientated towards the wound was determined at 10 h post wounding. Cells were fixed with 4% paraformaldehyde then co-stained with acetylated alpha tubulin and Giantin antibodies. Bar, 50 μm. The percent of cells at the wound edge having their Golgi apparatus in the forward-facing 120° sector was measured after wounding. Over 600 cells from 3 independent experiments were analyzed. Orientation of the Golgi apparatus with respect to the wound edge corresponds to percent on the ordinate. *, *P* < 0.05.

### Immunocytochemistry

Cells were seeded on glass coverslips pre-coated with fibronectin for defined time intervals. After that, cells were washed with PBS, then fixed with 4% paraformaldehyde for 10 min, and permeabilized with 0.15% Triton-×100 in PBS for 15 min and blocked with 5% BSA in PBS for 1 h at room temperature. Primary antibody diluted in PBS was added to the coverslips and incubated at 4 °C for overnight. Primary antibodies were used as follows: rabbit anti-paxillin (N-term) (1: 300, epitomics, Burlingame, USA), Rabbit anti-paxillin (phospho Y118) (1: 300, Abcam, Cambridge, UK), rabbit anti-zyxin (1:200, Epitomics, Burlingame, USA), Mouse anti-α-Actinin, clone BM-75.2, (1: 150,Sigam-Aldrich, St. Louis, USA), Mouse anti-Src (active), clone28 (1: 500,MBL international corporation),Rabbit anti-IQGAP1 (H-109) (1: 800, Santa Cruz Biotech, Dallas, USA), Rabbit anti-HECTD1(M03), clone 1E10 (1: 100, Abnova, Taipei, Taiwan), Mouse anti-Giantin (1:1000, a gift from Prof. Martin Spiess, Biozentrum, University of Basel). After washing the cells with PBS for 5 times with PBS, the secondary antibody (goat anti-rabbit-FITC, 1:1000; goat anti-mose-FITC, 1:1000; goat anti-mose-546, 1:1000, Invitrogen, Carlsbad, USA) tagged with fluorescent dye was added and incubated for 1 h in the dark at room temperature. After washing, cells were incubated in DAPI in PBS for 3 min at room temperature for counter staining. After washing, cells were mounted with Prolong® Gold Antifade Reagent and stored in 4 °C protected from light. The fluorescent pictures were made with the Nikon Confocal microscope.

### Western blot

Equal amounts of protein were loaded into the wells of SDS-PAGE gel, along with molecular weight markers. After running the gel at 100 V for 60–90 min, the protein was transferred to PVDF membrane and continued running at 300 mA for 60–80 min in pre-cooled transfer buffer. The blots were blocked in 5% milk in TTBS for 1 h at room temperature followed by primary antibody incubation for overnight at 4 °C. Primary antibodies were used as follows: rabbit anti-paxillin (N-term) (1: 300, epitomics, Burlingame, USA), Rabbit anti-paxillin (phospho Y118) (1: 300, Abcam, Cambridge, UK), rabbit anti-zyxin (1:200, Epitomics, Burlingame, USA), Rabbit anti-IQGAP1 (H-109) (1: 800, Santa Cruz Biotech, Dallas, USA), Rabbit GAPDH (14C10) (1:3000, Cell Signalling, Danvers, USA). After 3 times washing in TTBS, the blots were incubated in secondary antibody (goat anti-rabbit-HRP, 1:1000; goat anti-mose-HRP, 1:1000, Invitrogen, Carlsbad, USA) for 1 h at room temperature. To remove the unspecific bound antibody, the blots were washed in TTBS for 3 times. Bands were detected by ECL substrates, visualized by an infrared-based laser scanner (LiCor) and quantified using Image Lab software (Bio-Rad). The band intensity of wild-type cells of no stimulation was normalized with GAPDH as control and the other results were recorded as fold changes compared to control.

### Immunoprecipitation

Cell pellets were lysed with IP lysis buffer (20 mM Tris-HCl, PH 8.0, 137 mM NaCl, 1% NP40 and 2 mM EDTA supplemented with 1% protease inhibitor cocktail) on ice for 20 min and vortexed in between. Cellular débris was removed by centrifugation at 14,000 g for 5 min and the supernatant was transferred to pre-cooled fresh tubes. The protein amount was equilibrated with the IP buffer. 2 μl primary antibodies (Mouse anti-GFP GF28R, Thermo scientific, Waltham, USA) was added per 500 μg protein samples and incubated for overnight at 4 °C. The lysates were then incubated with prewashed protein A/G agarose beads (20 μl/500 μg protein) and rocked for 1 h at 4 °C. Beads were washed three times with IP buffer, 6000 rpm, 3 min. After washing, the beads were heated for 5 min at 95 °C in 2× Laemmli sample buffer. Target proteins were detected by western blot by using specific antibodies. Antibodies were used as: Rabbit anti-HECTD1 (M03), clone 1E10 (1:1000, Abnova, Taipei, Taiwan), Rabbit anti-PIP5K1A (1:1000, Cell Signaling, Danvers, USA), Rabbit anti-β-Catenin (D10A8) (1:1000, Cell Signaling, Danvers, USA).

### In vivo ubiquitination

MEF cells were transfected with plasmids DNA for HA-ubiquitin and GFP-IQGAP1 at ratio of 1:1. Twenty four hours after transfection, the cells were washed twice with PBS and changed to serum-free medium supplemented with 1 nM MG132 or DMSO, then incubated for overnight at 37 °C. For endogenous ubiquitination assay, MEF cells were seeded for 24 h and directly treated for starvation. Starvated cells were harvested as pellets and re-suspended in serum-free medium. Half of the pellets were spinned down and lysed with ubiquitination lysis buffer (50 mM Tris, pH 7.5, 1 mM EDTA, 150 mM NaCl, 0.1% Triton X-100, complete protease inhibitor cocktail, 100 μM MG132 and 100 μM N-ethylmalemide) on ice for 15 min followed with centrifugation (12,000 g, 5 min) at 4 °C. The other half was seeded on fibronectin pre-coated plates and cultivated in 37 °C for 60 min, after that, the plates were placed on ice, washed with pre-cooled PBS and lysed with lysis buffer (as previously) 15 min on ice before centrifugation. The supernatant was collected and then we continued with the protein concentration assay. Equal amount of protein was immune-precipitated with target protein and detection of ubiquitin by Western blot. Ubiquitination of target proteins were normalized by the protein amount in MEF cells.

### Statistical analysis

All data analyzed using the statistical software package SPSS 13.0 for Windows 7 (SPSS Inc., Chicago, Ill, USA). Normally distributed data was analyzed for statistical differences using the *t*-test (paired comparisons) or ANOVA (Analysis of Variance). For data not normally distributed, non-parametric ANOVA and the Mann-Whitney *U* test were used. All values are reported as means ± SEM. Differences are considered statistically significant with *P* < 0.05, highlighted with *. For each particular experiment, statistical analysis is presented in the figure legend.

## Results

### Loss of Hectd1 results in accelerating cell spreading/migration and impairs directional migration of cells

**Fig. 1:**
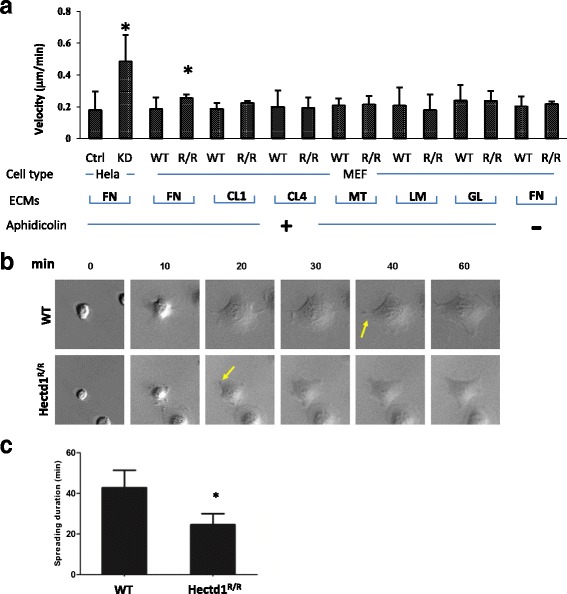
Fibronectin is a critical extracelluar matrix in HECTD1 regulating cell adhesion and the mutant HECTD1 accelerates cell spreading. **a** Wound healing assay. Equal amount of wild-type and *Hectd1* ^*R/R*^ MEF or Hela cells were seeded on 24 well plates coated with various ECMs for 24 h with 0.5% FBS, followed by starvation overnight with 1 μg/ml aphidicolin (see Methods). Wounds were created by 200 μl pipette tips and placed into a complete medium containing 10% FBS and aphidicolin. Migration images were acquired by time-lapse microscopy for 24 h. FN indicates fibronectin, CL1 stands for collagen type I, CL4 for collagen IV, MT for matrigel, LM for laminin and GL for gelatin. Experiments for each ECM were conducted for at least three times (paired *t* test, *P < 0.05). **b** Wild-type and *Hectd1* ^*R/R*^ cells were starved overnight, then plated on FN coated plates and immediately sent to time-lapse microscopy for recording 2 h (1 min / picture). Spreading on different time points were shown. **c** Duration of cell spreading was quantified by Image J software (paired *t* test, *P < 0.05)

Knockdown of HECTD1 by siRNAs in HeLa cells increased the rate of migration (Fig. 1a), to confirm this result we generated a mutant mouse of the E3 ubiquitin ligase for inhibin B receptor (Hectd1). We found that *Hectd1* homozygous mutant embryos display defective of neural tube closure with excencephaly (Additional file 1: Figure S1 and D’Alonzo et al., manuscript in preparation). We used mouse embryonic fibroblast (MEF) cells obtained from matched wild-type and *Hectd1* ^*R/R*^ mouse to analyze the time period from cell attachment to migration by time-lapse microscopy on various extracellular matrices, such as fibronectin (FN), collagen type I (CL1) or IV (CL4), matrigel (MT), laminin (LM) and gelatin (GL). There were significant differences in cell spreading and migration between the adhesion of wild-type and *Hectd1* ^*R/R*^ cells on FN but not or to much less extent on other ECMs (Fig. 1a), suggesting that HECTD1 regulates cell migration through only certain subtypes of integrin receptors.

**Fig. 2:**
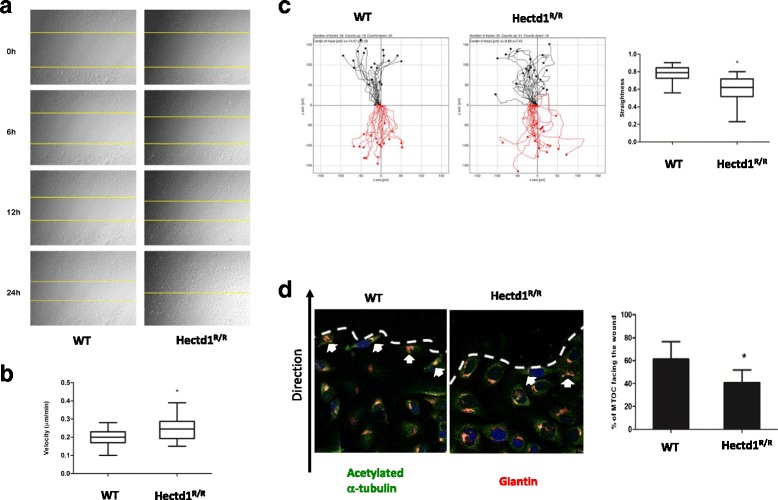
Mutant HECTD1 impairs directional cell migration. **a** Wound healing assays were performed on FN for 36 h in the medium containing 1 μM aphidicolin. **b** Velocity of cell migration was quantified by Image J software (paired *t* test, *P < 0.05). **c** Loss of HECTD1 impairs straightness of directional cell migration. Up and down plots of cell migration trajectories, velocity and straightness were measured by Manual tracking and chemotaxis tool (Image J). AU, arbitrary unit (paired *t* test, *P < 0.05). **d** Wound healing experiments were manipulated in 24 well plates with coverslips. The orientations of MTOC were indicated with white arrows. The percentage of MTOC orientated towards the wound was determined at 10 h post wounding (paired *t* test, *P < 0.05). Cells were fixed with 4% paraformaldehyde then co-stained with acetylated alpha tubulin and Giantin antibodies. Bar, 50 μm

When FN was used as an extracellular matrix, wild-type cells initially adopted a flattened morphology and started to form leading edges within 40 min while this process occurred approximately 10 min earlier in Hectd1 *Hectd1* ^*R/R*^ cells (Fig. 1b and c). We further examined the migration/directionality of cells in wound healing assays (Fig. 2). Loss of Hectd1 results in accelerating cell migration (Fig. 2a and time-lapse images were shown in Additional file 2: Figure S2A and Additional file 3: Figure S2B). The velocity (total distance/time) of Hectd1 *Hectd1* ^*R/R*^ cells was to 0.25 ± 0.07 μm/min compared to 0.19 ± 0.05 μm/min in wild-type cells (*P* < 0.05) (Fig. 2b), agreed to the results found in HeLa cells (Fig. 1a). Wild-type cells migrated in a cohesive fashion with little dispersion and with aligned displacement paths. In contrast, the trajectories of Hectd1 *Hectd1* ^*R/R*^ cells was more scattered (Fig. 2c).Additional file 2: Figure S2 Time lapse images for cell spreading and directional migration of *Hectd1* ^*R/R*^ cells. (AVI 36637 kb)
Additional file 3: Fiugre S2 Time lapse images for cell spreading and directional migration of *Hectd1* ^*R/R*^ cells. (AVI 36086 kb)

The straightness (Euclidean distance/Accumulated distance) was 0.60 ± 0.14 in *Hectd1* ^*R/R*^ cells versus 0.78 ± 0.09 in wild-type cells (*P* < 0.05) (Fig. 2c), indicating that the directed migration of cells was impaired. To further confirm the results, both wild-type and *Hectd1* ^*R/R*^ cells were stained with acetylated α tubulin and giantin (Fig. 2d), which are cell directional markers since the microtubule-organizing center (MTOC) and the Golgi matrix are reorient and toward leading edges during cell migration or wound healing [34–36]. The percentage of cells with giantin and acetylated α tubulin oriented to the wound was 61.29 ± 15.33% in wild-type cells, whereas this percentage dropped to 40.67 ± 11.25% in *Hectd1* ^*R/R*^ cells.

### Loss of Hectd1 impairs the subcellular localization of adhesion proteins

To dissect the molecular mechanism involved in causing the observed changes in cell migration in *Hectd1* ^*R/R*^ cells, we examined functional molecules in integrin signaling. α5β1 is the major FN receptor in fibroblasts but we did not observe differences in expression and localization of subunits α5 and β1, as well as β3 in contrast to the expression of α-actinin (Additional file 4: Figure S3 and data not shown), these results suggested that Hectd1 functions downstream of the receptors.

**Fig. 3:**
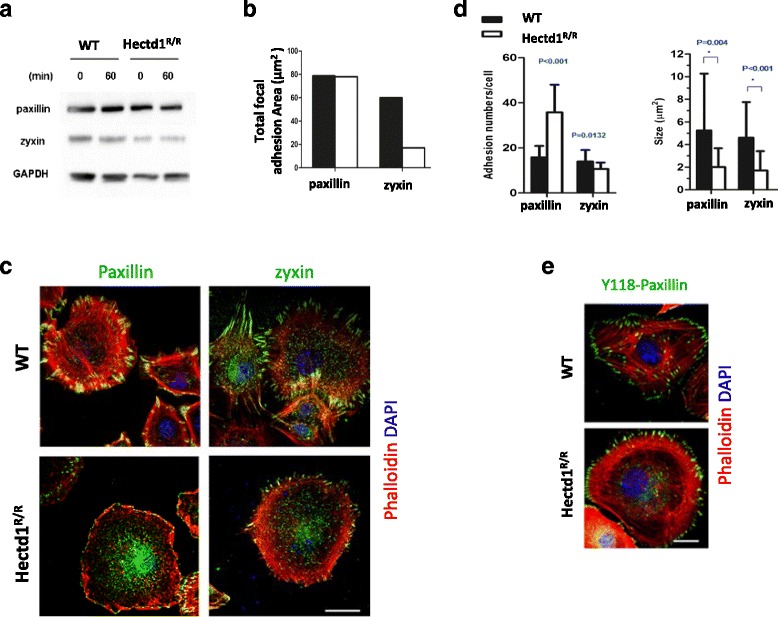
Loss of HECTD1 leads to mislocalization of paxillin and zyxin. **a** Expression of paxillin or zyxin was determined by Western Blots. **b** Total focal adhesion area was determined by Image J. **c** Wild-type and *Hectd1* ^*R/R*^ MEF cells were seeded on coverslips pre-coated with 1 μg/ml FN for 2 h, followed by anti-paxillin or anti-zyxin staining. Bar, 50 μm. **d** Amount of paxillin and zyxin and the individual focal adhesion were analyzed by Image J software. All the experiments were repeated at least 3 times and over 50 cells were analyzed in each group. Mann-Whitney *U* test were conducted. **e** In the same condition, cells were stained with anti-paxillin (phosphor Y118) and rhodamine phalloidin

The expression and localization of talin and vinculin, which have been shown to be incorporated into adhesive structures at early stage [37], did not significantly differ in both cell types when cultured on FN (data not shown). When both cells were cultured on FN, the total proteins of paxillin and zyxin were equally expressed (Fig. 3a) and the total focal adhesion area for paxillin did not have significantly different (Fig. 3b). However in *Hectd1* ^*R/R*^ cells, the proteins show a decrease of size distribution at the leading edges (Fig. 3d). Furthermore, paxillin-Y118, one of the FN-stimulated paxillin phosphorylation, became located in FAs where it was associated with stress fibers in WT. The expression of paxillin-Y118 was mostly located at the cell leading edges as FXs with disperse distribution in cytoplasm in *Hectd1* ^*R/R*^ cells (Fig. 3e). These results indicate that paxillin and zyxin were mislocalized in the adhesions of *Hectd1* ^*R/R*^ cells.

**Fig. 4:**
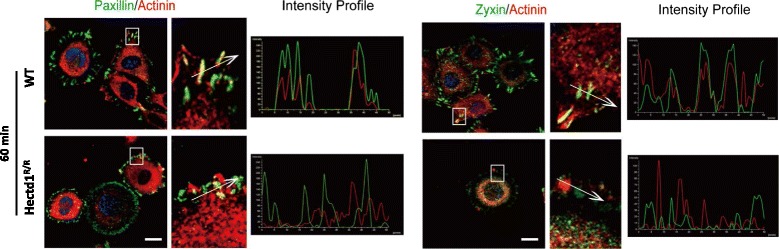
Loss of HECTD1 leads to mislocalization of α-actinin and paxillin/zyxin. Equal amounts of wild-type and HECTD1 MEF cells were seeded on culture dishes for 24 h, followed by starvation overnight. The cells plated on FN for 60 min were co-stained with anti-α-actinin and anti-paxillin or anti-zyxin, respectively. The dotted frame was zoomed out at the right panel, and the colocalization of two proteins across the dashed line was shown in the fluorescence intensity profiles. Bar, 20 μm

It has been suggested that α-actinin acts as a bridge to connect adhesion structures with the actin-cytoskeleton [38]. At the leading edges of wild-type cells activated by FN for 30, 60 and 90 min, α-actinin was mainly co-localized together with paxillin and zyxin in wild-type cells, while this co-localization was not present in *Hectd1* ^*R/R*^ cells. 60 min after spreading of *Hectd1* ^*R/R*^ cells, we could barely detect any patches of α-actinin at the cellular periphery. As the *Hectd1* ^*R/R*^ cells continued to migrate, some patches of α-actinin became visible at the leading edges, but still fewer than in wild-type cells (Fig. 4 and Additional file 5: Figure S4). These data indicate that Hectd1 exerts its function at adhesion sites.

### The formation of FAs but not FXs is impaired in *Hectd1*^*R/R*^ cells

**Fig. 5:**
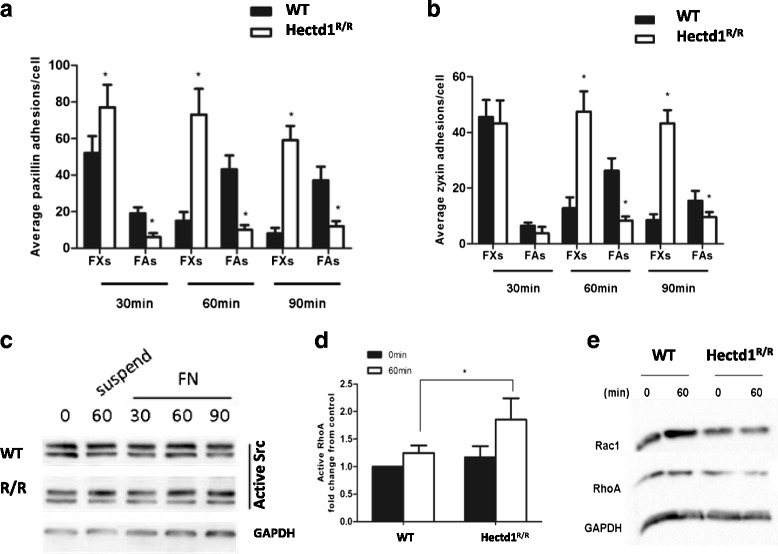
Loss of HECTD1 impairs formation of focal adhesions. Equal amounts of wild-type and *Hectd1* ^*R/R*^ MEF cells were seeded on FN coated coverslips for 30, 60 or 90 min. The cells were fixed and co-stained with phalloidin and anti-paxillin (**a**) or anti-zyxin (**b**), respectively. Average adhesions/cell and protein intensity were quantified by Lab Image software from 3 independent experiments, according to the sizes (FXs < = 1 μm^2^ [11], FAs > 2–5 μm^2^. paired *t* test, *P < 0.05). **c** Wild-type and *Hectd1* ^*R/R*^ cells were starved for overnight. cell lysates were either harvested immediately as 0 min control, suspended in culture medium at 37 °C for 60 min or plated on FN coated culture dishes for 30, 60 and 90 min at 37 °C. Lysates were analyzed by anti-Src (active) and GAPDH blotting. **d** After being starved for overnight, lysates of wild-type and *Hectd1* ^*R/R*^ cells were harvested immediately or plated on FN-coated culture dishes for 60 min at 37 °C. Activity of RhoA was measured by RhoA G-LISA Activation Assay Kit. Activity of RhoA was recorded as fold change from wild-type 0 min group based on three independent experiments (paired *t* test, *P < 0.05). **e** Expression of Rac1 and RhoA was determined by Western Blots

FXs are characterized as small punctate adhesions with <1 μm^2^ surface area lying close to the cell periphery, whereas FAs are classified as larger structures with their surface area varying between 5 and 20 μm^2^ [11]. Having allowed cell spreading for 30 min on FN, we started to analyze the dynamics of early (paxillin-only) versus late (paxillin-and-zyxin) adhesions. As shown in Fig. 5a, significantly more paxillin-containing FXs developed in *Hectd1* ^*R/R*^ cells (*P* < 0.05) than in wild-type cells. In contrast, FAs were more prominent in wild-type cells than in *Hectd1* ^*R/R*^ cells. However, zyxin, being a late-stage marker in adhesion formation, was similarly present in the FXs of both cell types (Fig. 5b). In migrating wild-type cells, both paxillin and zyxin showed similar distribution patterns in FAs after 60 min and after 90 min, whereas in *Hectd1* ^*R/R*^ cells the dominant cell adhesion structures consisted of FXs. Our results suggest that the defects in assembly of FAs at cell leading edges were caused by differences in the accumulation or transportation of proteins rather than by differences in the synthesis of the proteins.

One of the main kinases thought to be responsible for tyrosine phosphorylation of FA molecules is Src [39, 40]. Fig. 5c showed that there was no statistically significant difference in the expression level and activity of c-Src between *Hectd1* ^*R/R*^ and in wild-type cells after FN stimulation (*P* > 0.05).

Localized activation of Rac and Rho regulate adhesion dynamics during migration. Using the RhoA activation assay, we found that the activities of RhoA were significantly (*P* < 0.05) enhanced in *Hectd1* ^*R/R*^ cells 60 min after FN stimulation as compared that of wild-type cells (Fig. 5d), in which the total level of Rac1 and RhoA were not significant altered (Fig. 5e).

### IQGAP1 interacts and co-localizes with HECTD1

**Fig. 6:**
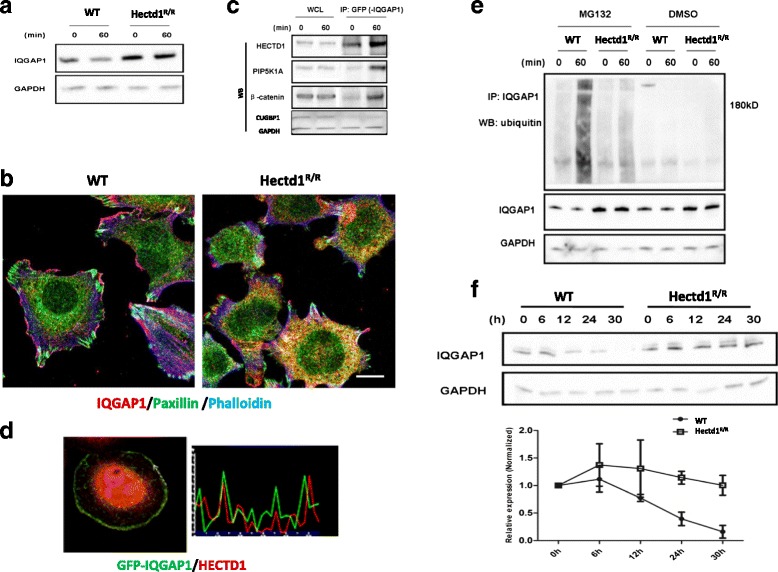
IQGAP1 interacts and colocalizes with HECTD1 in cell leading edge, and its ubiquitination is regulated by HECTD1. **a** Lysates of wild-type and *Hectd1* ^*R/R*^ MEF cells were harvested immediately or plated on FN coated culture dishes for 60 min at 37 °C and were analyzed by anti-IQGAP1 and GAPDH blotting. **b** Wild-type and *Hectd1* ^*R/R*^ MEF cells were stained with paxillin (*green*), IQGAP1 (*red*) and phalloidin (*blue*). **c** IQGAP1 interacts with HECTD1. HEK293 cells were stably transfected with GFP-IQGAP1 and seeded on fibronectin-coated dishes for 60 min, protein lysates were harvested and immunoprecipitated (IP) by GFP antibody. The lP lysates and whole cell lysates were used for detecting HECTD1, PIP5K1A and β-catenin by western blot. CUGBP1 served as a negative control. **d** Hela cell stably express His-HECTD1 was transiently transfected with GFP-IQGAP1 for 24 h. Cells were starved overnight and plated on FN-coated slides for 60 min, followed by fixation and staining with HECTD1. Note the site of colocalization shown in intensity profiles (white arrows). Pearson’s correlation coefficient was analyzed by Image J software. * *P* < 0.05. **e** Endogenous ubiquitination of IQGAP1. Wild-type and *Hectd1* ^*R/R*^ cells were treated with the proteasome inhibitor MG132 1 μg/ml or DMSO in serum starvation medium for overnight. Cell were lysed immediately or after 60 min seeded on FN coated dishes. The ubiquitination of IQGAP1 was further verified by immunoprecipitating IQGAP1 and detecting with an anti-ubiquitin antibody. **f** Half-life of IQGAP1 is increased in *Hectd1* ^*R/R*^ cells. Equal amounts of cells were plated on 100 mm dishes for 24 h and then treated with 100 μg/ml of Cycloheximide (CHX) for further 6 h (hours), 12 h, 24 h and 30 h. Cell pellets were harvested and the expression of IQGAP1 was detected by Western blot. Relative protein expression is quantified by densitometric analysis of Western blots with Image J software, based on three independent experiments

We found that IQGAP1 is a protein component of Hectd1 complexes [30] involved in formation of integrin adhesome and membrane ruffling. It has been demonstrated that IQGAP1 is an important factor in regulation of cell migration [26, 41]. As shown in Fig. 6a, the protein level of IQGAP1 was higher in *Hectd1* ^*R/R*^ cells than wild-type cells (*P* < 0.05). Consistent with this result, we observed that IQGAP1 is not only expressed in the leading edge of the *Hectd1* ^*R/R*^ cells but also heavily present in entire cytoplasm (Fig. 6b). Thus, we further focus on the functional relationship between IQGAP1 and HECTD1 in cell migration.

To confirm the interaction between HECTD1 and IQGAP1, we transfected GFP-IQGAP1 plasmids into HEK293 cells for immunoprecipitation. As shown in Fig. 6c, immunoprecipitation of endogenous HECTD1 resulted in the co-immunoprecipitation with GFP-IQGAP1 and co-immunoprecipitation was enhanced after 60 min of stimulation with FN. Next, we performed co-localization assays to verify the protein-protein interaction of IQGAP1 with HECTD1. HeLa cells transfected GFP-IQGAP1 were plated on FN coated plates for 60 min. Similar to the presence of HECTD1 in the cell, IQGAP1 was mainly localized in the cytoplasmic of the cells, but was enriched at the leading edge of cells. The Pearson’s correlation coefficient of GFP-IQGAP1 and HECTD1 at the cell leading edge was 0.65 ± 0.19 (Fig. 6d), suggesting that they co-localized with each other.

### Ubiquitination of IQGAP1 is regulated by HECTD1 and the half-life of IQGAP1 is increased in *Hectd1*^*R/R*^ cells

To evaluate whether IQGAP1 is ubiquitinated by HECTD1 we first examined the ubiquitination level of IQGAP1. We treated cells with the proteasome inhibitor MG132 to block the ubiquitin-proteasome degradation pathway. Compared to DMSO-treated control cells the overall ubiquitination level of IQGAP1was increased after treatment with MG132. The degree of ubiquitination of IQGAP1 after treatment with MG132 was more pronounced in wild-type cells than in *Hectd1* ^*R/R*^ cells 60 min after stimulation with FN (Fig. 6e).

We then verified whether the half-life of IQGAP1 varies accordingly in wild-type and in *Hectd1* ^*R/R*^ cells. We tested the degradation profile of IQGAP1 using cycloheximide (CHX-chase experiment). The CHX-chase experiments showed that the IQGAP1 level remained largely unchanged after up to 30 h in *Hectd1* ^*R/R*^ cells, whereas in wild-type cells this level decreased to near 50% within 12 h (Fig. 6f), suggesting that HECTD1 is involved in the degradation of IQGAP1.

### Overexpression of GFP-IQGAP1 in wild-type cells induces defects of FAs

**Fig. 7:**
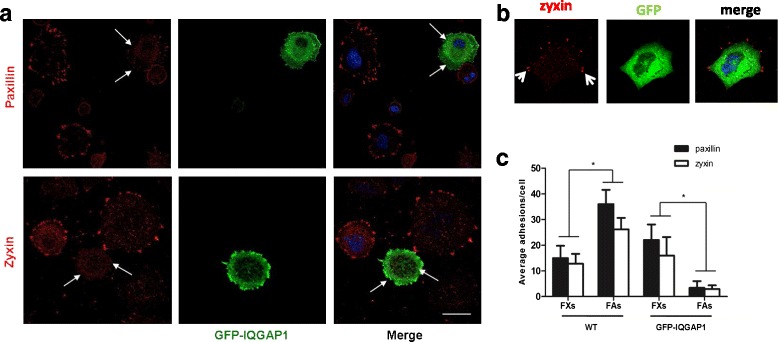
Overexpression of IQGAP1 affects focal adhesions formation. **a** Wild-type cells were transfected with GFP-IQGAP1 for 24 h. After starved overnight, cells were seeded on FN coated plate for 60 min and were subsequently fixed and stained with anti-paxillin and -zyxin, respectively. Non-transfected cells were used as control (arrow head). Note impaired formation of focal adhesions in GFP-IQGAP1 transfected cells (white arrows) compared with the control cells. **b** Quantification of average adhesions/cell is shown. Bar, 20 μm. *P < 0.05 (Mann-Whitney *U* test)

As IQGAP1 can be ubiquitinated by HECTD1 and degraded and as IQGAP1 has been reported to regulate FAs and cell migration [28], we speculated that the elevated protein level of IQGAP1 in *Hectd1* ^*R/R*^ cells were the direct cause of the impaired formation of FAs. In order to examine this hypothesis, we overexpressed GFP-IQGAP1 in wild-type cells, then performed immunostaining for paxillin and zyxin and measured the average number FXs and FAs per cell at different time points using paxillin or zyxin as markers. Interestingly, regardless whether paxillin or zyxin was chosen as the marker, the expression of FAs was dramatically decreased in the cells overexpressing GFP-IQGAP1 compared to non-transfected wild-type cells (Fig. 7a). In contrast, the expression of FAs in GFP expressing cells remained no change (Fig. 7b). In wild-type cell the ratio of FAs to FXs was 1/2, while the ratio of FAs to FXs decreased to around 1/8 in GFP-IQGAP1-overexpressed cells (Fig. 7c).

### Knockdown of IQGAP1 rescues the dynamics of FAs, the duration of cell spreading and directional cell migration in *Hectd1*^*R/R*^ cells

**Fig. 8:**
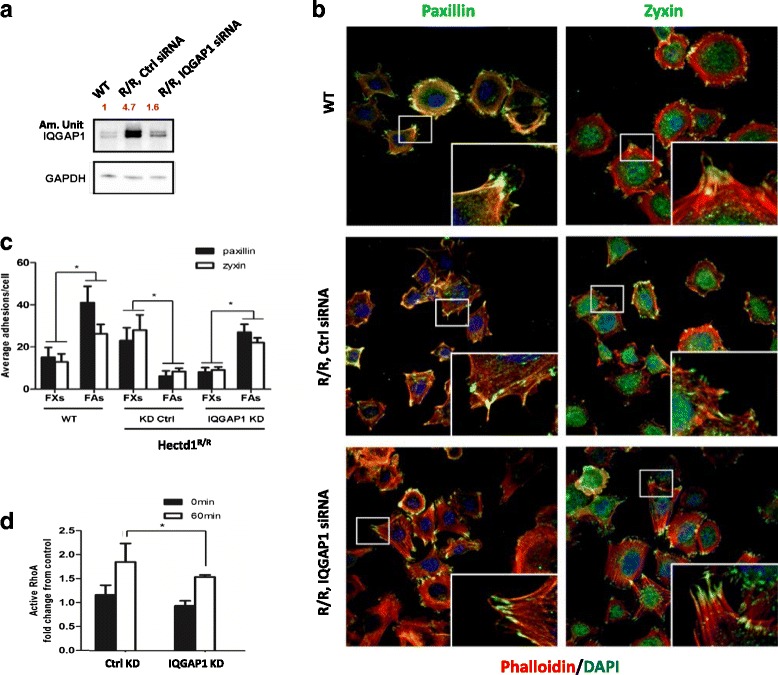
Knockdown of IQGAP1 rescue focal adhesion formation in *Hectd1* ^*R/R*^ cells. **a** *Hectd1* ^*R/R*^ cells were transfected with control siRNA or IQGAP1 siRNA. 36 h after transfection, cell pellets were harvested and the level of IQGAP1 was detected by western blot. GAPDH was taken as endogenous control. **b** Knockdown of IQGAP1 rescued the formation of cell adhesions. After 36 h of transfection of *Hectd1* ^*R/R*^ cells with control or IQGAP1 siRNA, cells were starved overnight and plated on FN coated coverslips for 60 min, followed by fixing and phalloidin and paxillin/zyxin costaining. **c** Quantification of average cell adhesions per cell. Bar, 50 μm. *, *P* < 0.05 (Mann-Whitney *U* test). **d** *Hectd1* ^*R/R*^ cells were transfected with IQGAP1 siRNA (IQGAP1 KD) and control siRNA (Ctrl KD) respectively. After 36 h, cells were starved overnight and plated on FN coated culture dishes for 60 min. The activity of RhoA was measured in protein lysates by RhoA G-LISA Activation Assay Kit. Activity of RhoA was recorded as fold change from wild-type 0 min group based on three independent experiments (paired *t* test, *P < 0.05)

To further test our hypothesis whether overexpression of IQGAP1 is involved in dysfunctional cell adhesion, spreading and migration in *Hectd1* ^*R/R*^ cells, we transfected *Hectd1* ^*R/R*^ cells with IQGAP1-siRNA (siIQ) or with control-siRNA. As a result the protein level of IQGAP11 in *Hectd1* ^*R/R*^-siIQ-transfected cells was knockdown (am. Unit 4.7 to 1.6 as compared to 1 in the wt cells, Fig. 8a).

Subsequently, after IQGAP1-knockdown in *Hectd1* ^*R/R*^ cells we analyzed the cytoskeleton and the FAs by immunostaining for actin, paxillin and zyxin. As shown in Fig. 8b, different structures of actin could be clearly distinguished, including stress fibers in the cell body, cortical F-actin enriched at the periphery and well-organized lamellipodia structures at the leading edge in wild-type cells. In contrast, in control siRNA-treated *Hectd1* ^*R/R*^ cells, stress fibers were less prominent than in wild-type cells and lamellipodia were difficult to detect. Importantly, the formation of lamellipodia was rescued by down-regulation of IQGAP1-siRNA in *Hectd1* ^*R/R*^ cells.

In line with our previous results, taking paxillin and zyxin as cell adhesion markers, the ratio of FAs to FXs was about twice in wild-type cells, while in control siRNA-treated *Hectd1* ^*R/R*^ cells, the average ratio of the number of FAs/FXs fell to around 1/3. The ratio of FXs to FAs was rescued by IQGAP1-siRNA knockdown in *Hectd1* ^*R/R*^ cells, in which the FAs accounted for the majority of cell adhesions and the ratio of FAs to FXs per cell again became threefold (Fig. 8c). Moreover, activity of RhoA was also evidently increased in control siRNA Hectd1 mutant MEFs after FN stimulation for 60 min (Fig. 5d), whereas RhoA activity could be significantly inhibited by IQGAP1 siRNA scilencing in *Hectd1* ^*R/R*^ MEFs (*P* < 0.05) (Fig. 8d). These results suggest that in the absence of Hectd1, the activation of RhoA correlated with increased protein levels of IQGAP1.

**Fig. 9:**
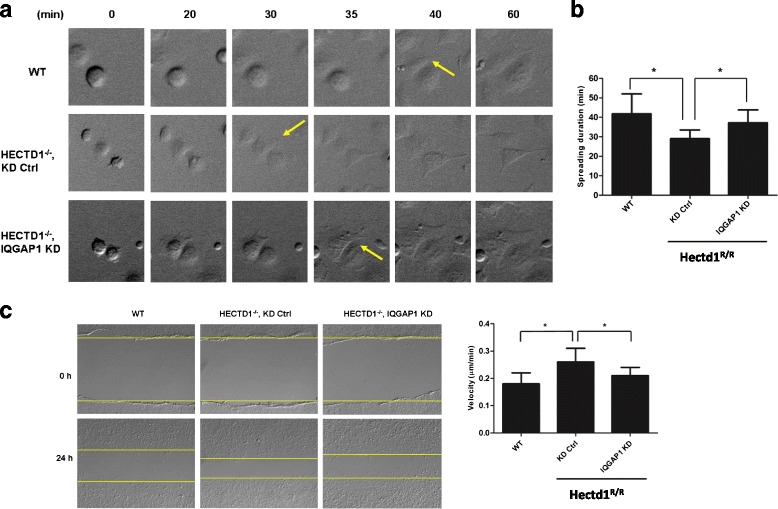
Knockdown of IQGAP1 rescue the defects of spreading and migration of *Hectd1* ^*R/R*^ cells. **a** After 36 h of transfection, cells were starved overnight and plated on FN-coated cell culture dishes and immediately sent to time-lapse recording for 2 h (1 min/picture). Spreading pictures at different time points were shown. Note for cells with leading protrusion (yellow arrows). **b** Quantification of duration of cell spreading on 30 min is shown. AU, arbitrary unit. *, paired *t* test, *P* < 0.05. **c** 24 h after siRNA transfection, wound healing assays were performed. Migration images were acquired by time-lapse microscopy for 24 h. The images were analyzed quantitatively by Image J software (paired *t* test, *P* < 0.05)

Furthermore we found that the spreading duration time shortened to (29.03 ± 4.48 min) in *Hectd1* ^*R/R*^ cells (*Hectd1* ^*R/R*^ control group) in contrast with (41.80 ± 10.19 min) in wild-type cells, and the spreading duration time in IQGAP1-silenced cells (37.23 ± 6.60 min) was partly rescued as compared to control siRNA-transfected cells (*P* < 0.05, *P* < 0.05, resp.) (Fig. 9a and b).

Next, in order to further investigate whether down-regulation of IQGAP1 in *Hectd1* ^*R/R*^ cells would also affect directional cell migration, confluent cell layers of wild-type cells and *Hectd1* ^*R/R*^ cells with down-regulated IQGAP1 through siRNA and *Hectd1* ^*R/R*^ cells with control siRNA were scratched and wound closure was recorded by time lapse microscopy. The migration speed of control-siRNA treated cells was 0.97 ± 0.14 μm/min, as compared with 0.86 ± 0.17 μm/min in wild-type cells, which was consistent with our previous results. The migration defect was rescued by siRNA-mediated down-regulation of IQGAP1 (0.94 ± 0.14 μm/min). Similarly, as compared with wild-type cells the straightness of directional cell migration was impaired in control-siRNA MEFs, whereas of siRNA-mediated knockdown of IQGAP1 compensated the defect (Fig. 9c).

## Discussion

Although the eminent role of HECTD1 in embryogenesis, including neural tube formation, placenta formation and embryonic growth, has been clearly demonstrated in at least two transgenic mouse models, limited information has been collected so far to uncover the regulatory mechanisms involved. Moreover, the involvement of HECTD1 in regulating cell migration during organogenesis has as yet remained unexplored. We observed that loss of HECTD1 induced earlier cell spreading and enhanced cell migration through controlling IQGAP1 and adhesion proteins. Our study proposes a new mechanism of HECTD1 in maintaining accurate cell movement during embryogenesis.

### HECTD1 is a selective effector of ECM-integrin signaling

The complexity of the molecular signaling responsible for ECM selective guidance is associated with various ligand-binding possibilities for integrin subtypes [42–45]. Our first observation was that the migration patterns of *Hectd1* ^*R/R*^ cells is significantly different to that of wild-type cells on various ECM when these cells were incubated in culture medium lacking serum. These results indicate that factors in serum may compensate the loss of HECTD1 through yet unknown signaling pathways. In addition, the localization of paxillin and zyxin but not of talin and vinculin was different during migration of these cells on FN. Furthermore, more FAs formed in wild-type cells whereas more FXs developed in mutant cells. These differences were not apparent when the cells were cultured on collagen type I and on gelatin.

The adhesion proteins are known to have a specific binding relationship with integrins. For instance, paxillin is normally binds to ɑ_5_β_1_, ɑ_4_β_1_, ɑ_5_β_3_ and ɑ_4_β_3_ integrins [46–49], while zyxin binds to the integrin subtypes ɑ_5_β_1_ and ɑ_5_β_5_ [50, 51]. Besides, the integrin motifs expressed in different cell types may vary considerably. For example, in fibroblasts ɑ_1_β_1_, ɑ_2_β_1_, ɑ_3_β_1_ and ɑ_5_β_1_ integrins were predominantly identified [52]. Thus, our results suggest that the regulation of the composition of FAs and FXs by HECTD1 in fibroblasts depends on specific integrin receptor subtypes, probably via coordinating with ɑ_5_β_1_.

### The involvement of IQGAP1 in regulating adhesion dynamics is mediated by HECTD1

IQGAP1 has been widely reported to be involved in regulating FAs dynamics and cell migration. We confirmed the interaction of HECTD1 with IQGAP1 and their co-localization through co-immunoprecipitation and double-labeled immunocytochemistry, respectively. We observed that loss of HECTD1, being an E3-ubiquitin ligase, enhances the protein level of IQGAP1 through decreased ubiquitination. When IQGAP1 was overexpressed in wild-type cells, it reduced the formation of FAs as determined by differences in the expression of paxillin and zyxin. Moreover, siRNA knockdown of IQGAP1 in *Hectd1* ^*R/R*^ cells compensated the defects in the formation of cell adhesions, in cell spreading and migration. Taken all these results together, IQGAP1 has now been demonstrated to be regulated through degradation by HECTD1. We therefore conclude that HECTD1 regulates cell adhesion and controls cell spreading and migration via IQGAP1.

### High FXs-FAs ratio in *Hectd1*^*R/R*^ cells contributes to higher motility

We have demonstrated that the mutation of HECTD1 results in altered cell spreading and migration, in which the velocity of *Hectd1* ^*R/R*^ cells was increased with impaired directionality. In our assay, we used aphidicolin to ensure that proliferation did not interfere with cell migration. We also showed that HECTD1 ablation did not influence cell migration speed in the presence of 10% FBS without aphidicolin in MEF cells on FN. This result is consistent with Li’s result [18], in which 10 ng/ml of EGF was used in breast cancer cells. We therefore, used the same setting for the Hectd1/IQGAP1 double knockout/down experiments. Instead of measuring total adhesion structures we differentiated FXs from FAs in cells. Interestingly, when compared to wild-type cells, the average total number of small adhesions in *Hectd1* ^*R/R*^ cells is increased. Moreover, FXs are evidently associated with fewer FAs, which are bigger in size than FXs in *Hectd1* ^*R/R*^ cells than in their wild-type counterparts. Maturation of adhesions occurs along an α-actinin-actin template that elongates centripetally from nascent adhesions. We found that α-actinin is co-localized with paxillin or zyxin at the leading edge of wild-type cells, but not in *Hectd1* ^*R/R*^ cells. These results suggest that in *Hectd1* ^*R/R*^ cells, FXs including paxillin fail to reassemble or/and cannot mature to FAs.

Since the presence of FXs and nascent adhesions is a marker of highly motile cells, their quick appearance and turnover correlate directly with protrusion and cell movement. The higher number of small paxillin patches in *Hectd1* ^*R/R*^ cells strongly correlates with their increased motility and fast spreading. In motile cells, the recruitment of the adhesion proteins into FXs occurs sequentially, so that composition of the specific proteins relies on their age. Moreover, using double color staining, time-lapse assay, one study demonstrated that the transition from paxillin-rich FXs to zyxin-containing FAs takes place after the leading edge stops advancing or retracts [37]. Generally, zyxin has been thought to be a component of FA plaques and is absent from FXs [37, 53]. Although these three types of adhesions are distinguishable, there is always a continuum between types and many of the same adhesion proteins have been identified in each [54].

Consistent with our findings, in highly motile cells such as melanoma cells, glioma cells and growing neurons [55] the dynamic adhesions most similar to FXs are enriched in the leading edge of cells and act as common features of rapid cell movement [56]. Therefore, we conclude that the accumulation of paxillin and zyxin in the lamellipodia of FXs is a major hallmark of highly motile cells. Thompson has also proved that decreased size of FAs is related to higher velocity and impaired directionality of cells, and vice versa [57]. Increased numbers of the adhesions are accompanied with a lesser motility [58, 59]. Here, we show that the dynamics of cell adhesion are responsible for the velocity of cells during migration.

We propose that 30 min spreading is too early for the recruitment of abundant zyxin into FAs, so that the presence of zyxin is not enough to distinguish the difference in *Hectd1* ^*R/R*^ and wild-type cells.

### Model for the role of HECTD1 in regulating cell movement

**Fig. 10:**
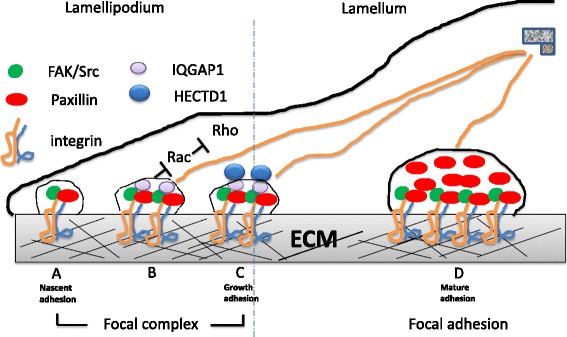
The role of HECTD1 in FAs formation. Upon binding of integrins to ECMs (e.g., Fibronectin), FAK/src signal pathway is activated and the recruitment of paxillin to the binding sites results in phosphorylation of paxillin at Y118 and the initiation of Focal complexs formation (**a**). Subsequently further recruitment of IQGAP1 passes the activation signals to small GTPases, such as Rac1 and RhoA. Together with FLAm, IQGAP1 inhibits Rac1 activity (**b**). The role of IQGAP1 on Rac/Rho is regulated by HECTD1 (**c**). Removal of IQGAP1 from Focal complexs triggers the maturation of focal adhesions by recruiting more paxillin and zyxin (**d**). HECTD1 is a key regulator of IQGAP1 and through this interaction HEDTD1 impacts on cellular adhesion and movement

Our data revealed that FXs in *Hectd1* ^*R/R*^ cells failed to recruit enough adhesion proteins (such as paxillin and zyxin) to mature into FAs. Therefore, the alteration in number and/or size of FXs is expected to influence cell motility. Thus, we propose the following model for the role of HECTD1 in cell movement (Fig. 10).

During cell spreading and early migration the cell receives stimulating signals from its extracellular environment, such as FN in the extracellular matrix, which activates relevant integrin receptors and Src in the cell leading edge. The recruitment of paxillin results in phosphorylation of paxillin at Y118. With this event the initiation of focal complexs formation becomes complete. The activation signals are passed to small GTPases, such as Rac1 and RhoA via IQGAP1 recruitment. Together with filamin-A, IQGAP1 inhibits Rac1 activity [60]. Subsequently removal of IQGAP1 from Focal complexs together with high RhoA activities triggers the maturation of focal adhesions by recruiting more paxillin and zyxin.

As an E3 ubiquitin ligase, HECTD1 regulates the level of IQGAP1 through ubquitination. Loss of HECTD1 prolonges the half-life of IQGAP1 and thereby reduces the recruitment of paxillin and zyxin. As a result, FXs failed to mature into FAs through a deficiency of paxillin and zyxin. HECTD1 is a key regulator of IQGAP1 and through this interaction HEDTD1 impacts on cellular adhesion and movement.

## Conclusions

By generating a knockout mouse of the E3 ubiquitin ligase for inhibin B receptor (HECTD1), we reveal a molecular mechanism in which IQGAP1 is one of the effectors of HECTD1. HECTD1 interacted with IQGAP1 and regulated its degradation through ubiquitination. Increased expression of IQGAP1 in *Hectd1* ^*R/R*^ cells lead to the mis-localized paxillin and zyxin so that the formation of focal adhesions (FAs) was impaired, resulting in accelerated cell spreading and migration but impaired directionality of migration.

## Additional files

Additional file 1: Fiugre S1. Generation of *Hectd1* mutant animals. (PPTX 142 kb)
Additional file 4: Figure S3. Subcellular localization of adhesion proteins in *Hectd1* ^*R/R*^ cells. (PPTX 1221 kb)
Additional file 5: Figure S4. Loss of HECTD1 leads to mislocalization of α-actinin and paxillin/zyxin. (PPTX 1571 kb).

## Abbreviations

APC: Adenomatous polyposis coli
CL1: Collagen type I
CL4: Collagen type IV
CSK: C-terminal Src kinase
ECM: Extracellular matrix
FAK: Focal adhesion kinase
FAs: Focal adhesions
FN: Fibronectin
FXs: Focal complexes
GL: Gelatin
LacZ: β-galactosidase activity
LM: Laminin
MEF: Mouse embryonic fibroblasts
MT: Matrigel
MTOC: Microtubule-organizing center
PIP5K: Phosphatidylinositol 4-phosphate 5-kinase

## References

[1] Tada M, Heisenberg CP (2012). Convergent extension: using collective cell migration and cell intercalation to shape embryos. Development.

[2] Dzamba BJ, Jakab KR, Marsden M, Schwartz MA, DeSimone DW (2009). Cadherin adhesion, tissue tension, and noncanonical Wnt signaling regulate fibronectin matrix organization. Dev Cell.

[3] Theveneau E, Mayor R (2012). Cadherins in collective cell migration of mesenchymal cells. Curr Opin Cell Biol.

[4] Weijer CJ (2009). Collective cell migration in development. J Cell Sci.

[5] Lauffenburger DA, Horwitz AF (1996). Cell migration: a physically integrated molecular process. Cell.

[6] Chamaraux F, Ali O, Keller S, Bruckert F, Fourcade B (2008). Physical model for membrane protrusions during spreading. Phys Biol.

[7] Serrels B, Serrels A, Brunton VG, Holt M, McLean GW, Gray CH (2007). Focal adhesion kinase controls actin assembly via a FERM-mediated interaction with the Arp2/3 complex. Nat Cell Biol.

[8] Loosli Y, Luginbuehl R, Snedeker JG (2010). Cytoskeleton reorganization of spreading cells on micro-patterned islands: a functional model. Philos Trans A Math Phys Eng Sci.

[9] Wakatsuki T, Wysolmerski RB, Elson EL (2003). Mechanics of cell spreading: role of myosin II. J Cell Sci.

[10] Izzard CS, Lochner LR (1976). Cell-to-substrate contacts in living fibroblasts: an interference reflexion study with an evaluation of the technique. J Cell Sci.

[11] Galbraith CG, Yamada KM, Sheetz MP (2002). The relationship between force and focal complex development. J Cell Biol.

[12] Zaidel-Bar R, Itzkovitz S, Ma’ayan A, Iyengar R, Geiger B (2007). Functional atlas of the integrin adhesome. Nat Cell Biol.

[13] Zhang H. Homo sapiens E3 ligase for inhibin receptor mRNA, complete cds http://www.ncbi.nlm.nih.gov/nuccore/AY254380.1. Accessed 22 Apr 2003.

[14] Sarkar AA, Zohn IE (2012). Hectd1 regulates intracellular localization and secretion of Hsp90 to control cellular behavior of the cranial mesenchyme. J Cell Biol.

[15] Tran H, Bustos D, Yeh R, Rubinfeld B, Lam C, Shriver S (2013). HectD1 E3 ligase modifies adenomatous polyposis coli (APC) with polyubiquitin to promote the APC-axin interaction. J Biol Chem.

[16] Zohn IE, Anderson KV, Niswander L (2007). The Hectd1 ubiquitin ligase is required for development of the head mesenchyme and neural tube closure. Dev Biol.

[17] Sarkar AA, Nuwayhid SJ, Maynard T, Ghandchi F, Hill JT, Lamantia AS (2014). Hectd1 is required for development of the junctional zone of the placenta. Dev Biol.

[18] Li X, Zhou Q, Sunkara M, Kutys ML, Wu Z, Rychahou P (2013). Ubiquitylation of phosphatidylinositol 4-phosphate 5-kinase type I gamma by HECTD1 regulates focal adhesion dynamics and cell migration. J Cell Sci.

[19] Brill S, Li S, Lyman CW, Church DM, Wasmuth JJ, Weissbach L (1996). The Ras GTPase-activating-protein-related human protein IQGAP2 harbors a potential actin binding domain and interacts with calmodulin and Rho family GTPases. Mol Cell Biol.

[20] Hart MJ, Callow MG, Souza B, Polakis P (1996). IQGAP1, a calmodulin-binding protein with a rasGAP-related domain, is a potential effector for cdc42Hs. EMBO J.

[21] Kuroda S, Fukata M, Kobayashi K, Nakafuku M, Nomura N, Iwamatsu A (1996). Identification of IQGAP as a putative target for the small GTPases, Cdc42 and Rac1. J Biol Chem.

[22] Mateer SC, Wang N, Bloom GS (2003). IQGAPs: integrators of the cytoskeleton, cell adhesion machinery, and signaling networks. Cell Motil Cytoskeleton.

[23] Fukata M, Watanabe T, Noritake J, Nakagawa M, Yamaga M, Kuroda S (2002). Rac1 and Cdc42 capture microtubules through IQGAP1 and CLIP-170. Cell.

[24] Watanabe T, Wang S, Noritake J, Sato K, Fukata M, Takefuji M (2004). Interaction with IQGAP1 links APC to Rac1, Cdc42, and actin filaments during cell polarization and migration. Dev Cell.

[25] Brown MD, Sacks DB (2006). IQGAP1 in cellular signaling: bridging the GAP. Trends Cell Biol.

[26] Choi S, Thapa N, Hedman AC, Li Z, Sacks DB, Anderson RA (2013). IQGAP1 is a novel phosphatidylinositol 4,5 bisphosphate effector in regulation of directional cell migration. EMBO J.

[27] Liu C, Billadeau DD, Abdelhakim H, Leof E, Kaibuchi K, Bernabeu C (2013). IQGAP1 suppresses TbetaRII-mediated myofibroblastic activation and metastatic growth in liver. J Clin Invest.

[28] Noritake J, Watanabe T, Sato K, Wang S, Kaibuchi K (2005). IQGAP1: a key regulator of adhesion and migration. J Cell Sci.

[29] Kuo JC, Han X, Hsiao CT, Yates JR, Waterman CM (2011). Analysis of the myosin-II-responsive focal adhesion proteome reveals a role for beta-Pix in negative regulation of focal adhesion maturation. Nat Cell Biol.

[30] Schiller HB, Friedel CC, Boulegue C, Fassler R (2011). Quantitative proteomics of the integrin adhesome show a myosin II-dependent recruitment of LIM domain proteins. EMBO Rep.

[31] Wickstrom SA, Lange A, Hess MW, Polleux J, Spatz JP, Kruger M (2010). Integrin-linked kinase controls microtubule dynamics required for plasma membrane targeting of caveolae. Dev Cell.

[32] Schiefermeier N, Scheffler JM, de Araujo ME, Stasyk T, Yordanov T, Ebner HL (2014). The late endosomal p14-MP1 (LAMTOR2/3) complex regulates focal adhesion dynamics during cell migration. J Cell Biol.

[33] Stanford WL, Cohn JB, Cordes SP (2001). Gene-trap mutagenesis: past, present and beyond. Nat Rev Genet.

[34] Euteneuer U, Schliwa M (1992). Mechanism of centrosome positioning during the wound response in BSC-1 cells. J Cell Biol.

[35] Schaar BT, McConnell SK (2005). Cytoskeletal coordination during neuronal migration. Proc Natl Acad Sci U S A.

[36] Farhan H, Wendeler MW, Mitrovic S, Fava E, Silberberg Y, Sharan R (2010). MAPK signaling to the early secretory pathway revealed by kinase/phosphatase functional screening. J Cell Biol.

[37] Zaidel-Bar R, Ballestrem C, Kam Z, Geiger B (2003). Early molecular events in the assembly of matrix adhesions at the leading edge of migrating cells. J Cell Sci.

[38] Maruyama K, Ebashi S (1965). Alpha-actinin, a new structural protein from striated muscle. II. Action on actin. J Biochem.

[39] Frame MC (2004). Newest findings on the oldest oncogene; how activated src does it. J Cell Sci.

[40] Schlaepfer DD, Mitra SK (2004). Multiple connections link FAK to cell motility and invasion. Curr Opin Genet Dev.

[41] Jacquemet G, Humphries MJ (2013). IQGAP1 is a key node within the small GTPase network. Small GTPases.

[42] Doughman RL, Firestone AJ, Wojtasiak ML, Bunce MW, Anderson RA (2003). Membrane ruffling requires coordination between type Ialpha phosphatidylinositol phosphate kinase and Rac signaling. J Biol Chem.

[43] Dall’Armi C, Devereaux KA, Di Paolo G (2013). The role of lipids in the control of autophagy. Curr Biol.

[44] Barczyk M, Carracedo S, Gullberg D (2010). Integrins. Cell Tissue Res.

[45] Levental KR, Yu H, Kass L, Lakins JN, Egeblad M, Erler JT (2009). Matrix crosslinking forces tumor progression by enhancing integrin signaling. Cell.

[46] Crowe DL, Ohannessian A (2004). Recruitment of focal adhesion kinase and paxillin to beta1 integrin promotes cancer cell migration via mitogen activated protein kinase activation. BMC Cancer.

[47] Laukaitis CM, Webb DJ, Donais K, Horwitz AF (2001). Differential dynamics of alpha 5 integrin, paxillin, and alpha-actinin during formation and disassembly of adhesions in migrating cells. J Cell Biol.

[48] Schaller MD, Otey CA, Hildebrand JD, Parsons JT (1995). Focal adhesion kinase and paxillin bind to peptides mimicking beta integrin cytoplasmic domains. J Cell Biol.

[49] Schaller MD, Parsons JT (1995). pp125FAK-dependent tyrosine phosphorylation of paxillin creates a high-affinity binding site for Crk. Mol Cell Biol.

[50] Bianchi-Smiraglia A, Kunnev D, Limoge M, Lee A, Beckerle MC, Bakin AV (2013). Integrin-beta5 and zyxin mediate formation of ventral stress fibers in response to transforming growth factor beta. Cell Cycle.

[51] Mise N, Savai R, Yu H, Schwarz J, Kaminski N, Eickelberg O (2012). Zyxin is a transforming growth factor-beta (TGF-beta)/Smad3 target gene that regulates lung cancer cell motility via integrin alpha5beta1. J Biol Chem.

[52] Mineur P, Guignandon A, Lambert Ch A, Amblard M, Lapiere Ch M, Nusgens BV (2005). RGDS and DGEA-induced [Ca2+]i signalling in human dermal fibroblasts. Biochim Biophys Acta.

[53] Zaidel-Bar R, Cohen M, Addadi L, Geiger B (2004). Hierarchical assembly of cell-matrix adhesion complexes. Biochem Soc Trans.

[54] Parsons JT, Horwitz AR, Schwartz MA (2010). Cell adhesion: integrating cytoskeletal dynamics and cellular tension. Nat Rev Mol Cell Biol.

[55] Gatlin JC, Estrada-Bernal A, Sanford SD, Pfenninger KH (2006). Myristoylated, alanine-rich C-kinase substrate phosphorylation regulates growth cone adhesion and pathfinding. Mol Biol Cell.

[56] Estrada-Bernal A, Gatlin JC, Sunpaweravong S, Pfenninger KH (2009). Dynamic adhesions and MARCKS in melanoma cells. J Cell Sci.

[57] Thompson O, Moore CJ, Hussaina SA, Kleino I, Peckham M, Hohenester E (2010). Modulation of cell spreading and cell-substrate adhesion dynamics by dystroglycan. J Cell Sci.

[58] Lu ZM, Jiang GQ, Blume-Jensen P, Hunter T (2001). Epidermal growth factor-induced tumor cell invasion and metastasis initiated by dephosphorylation and downregulation of focal adhesion kinase. Mol Cell Biol.

[59] Nagano M, Hoshino D, Koshikawa N, Akizawa T, Seiki M (2012). Turnover of focal adhesions and cancer cell migration. Int J Cell Biol.

[60] Jacquemet G, Morgan MR, Byron A, Humphries JD, Choi CK, Chen CS (2013). Rac1 is deactivated at integrin activation sites through an IQGAP1-filamin-A-RacGAP1 pathway. J Cell Sci.

