# Supplementary material for: HECTD1 controls the protein level of IQGAP1 to regulate the dynamics of adhesive structures
Source: Cell Commun Signal. 2017 Jan 5;15:2. doi: 10.1186/s12964-016-0156-8 (PMC5225595; doi:10.1186/s12964-016-0156-8)
Supplement: Additional file 1: Fiugre S1. — Generation of Hectd1 mutant animals. (PPTX 142 kb) [file 12964_2016_156_MOESM1_ESM.pptx]

## Slide 1
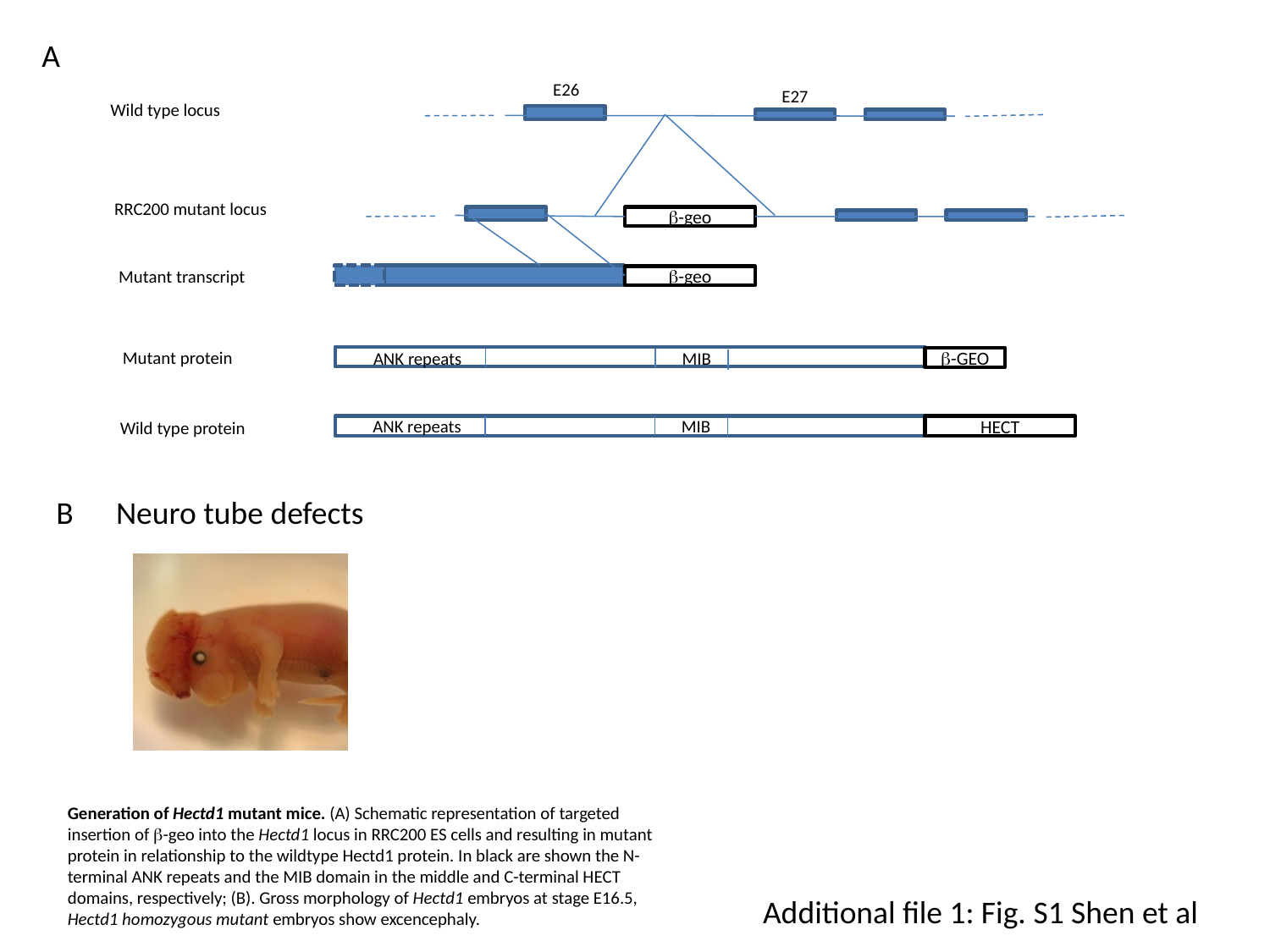

A
E26
E27
Wild type locus
RRC200 mutant locus
b-geo
Mutant transcript
b-geo
Mutant protein
ANK repeats
MIB
b-GEO
ANK repeats
MIB
Wild type protein
HECT
B
Neuro tube defects
Generation of Hectd1 mutant mice. (A) Schematic representation of targeted insertion of b-geo into the Hectd1 locus in RRC200 ES cells and resulting in mutant protein in relationship to the wildtype Hectd1 protein. In black are shown the N-terminal ANK repeats and the MIB domain in the middle and C-terminal HECT domains, respectively; (B). Gross morphology of Hectd1 embryos at stage E16.5, Hectd1 homozygous mutant embryos show excencephaly.
Additional file 1: Fig. S1 Shen et al
