# Supplementary material for: HECTD1 controls the protein level of IQGAP1 to regulate the dynamics of adhesive structures
Source: Cell Commun Signal. 2017 Jan 5;15:2. doi: 10.1186/s12964-016-0156-8 (PMC5225595; doi:10.1186/s12964-016-0156-8)
Supplement: Additional file 4: Figure S3. — Subcellular localization of adhesion proteins in Hectd1 R/R cells. (PPTX 1221 kb) [file 12964_2016_156_MOESM4_ESM.pptx]

## Slide 1
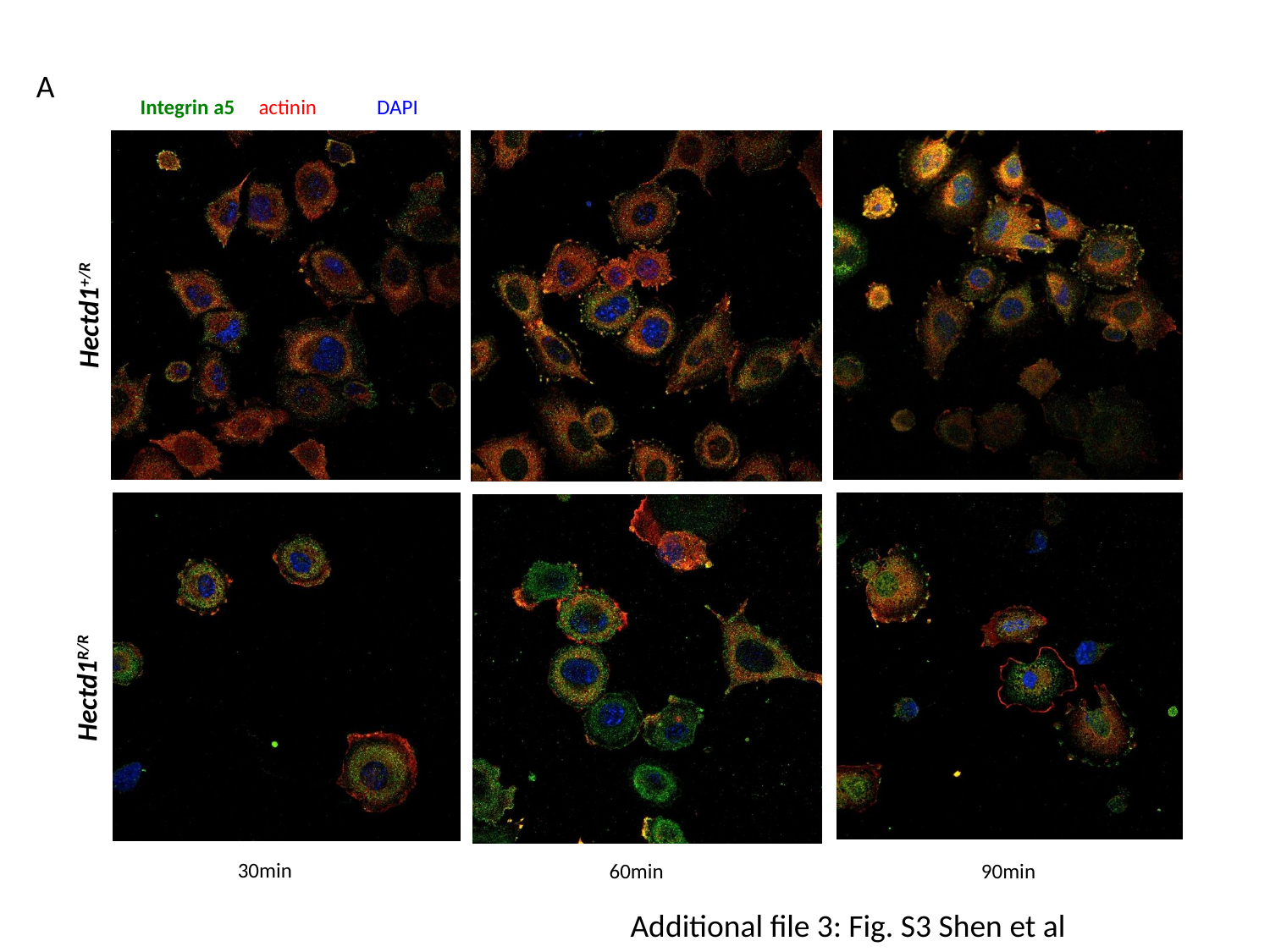

A
Integrin a5
actinin
DAPI
Hectd1+/R
Hectd1R/R
30min
60min
90min
Additional file 3: Fig. S3 Shen et al

## Slide 2
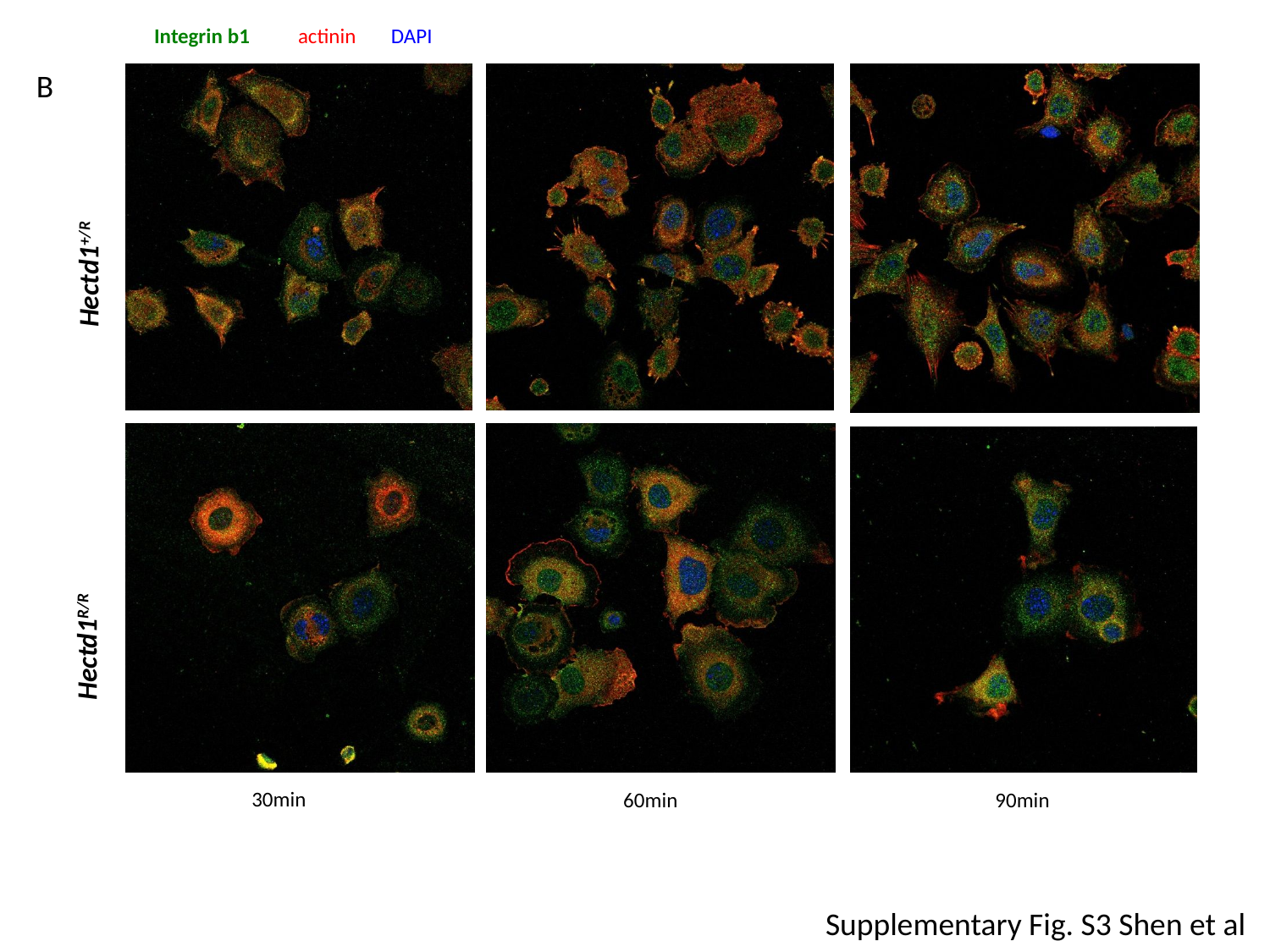

Integrin b1
actinin
DAPI
30min
60min
90min
B
Hectd1+/R
Hectd1R/R
Supplementary Fig. S3 Shen et al
