# Supplementary material for: HECTD1 controls the protein level of IQGAP1 to regulate the dynamics of adhesive structures
Source: Cell Commun Signal. 2017 Jan 5;15:2. doi: 10.1186/s12964-016-0156-8 (PMC5225595; doi:10.1186/s12964-016-0156-8)
Supplement: Additional file 5: Figure S4. — Loss of HECTD1 leads to mislocalization of α-actinin and paxillin/zyxin. (PPTX 1571 kb). [file 12964_2016_156_MOESM5_ESM.pptx]

## Slide 1
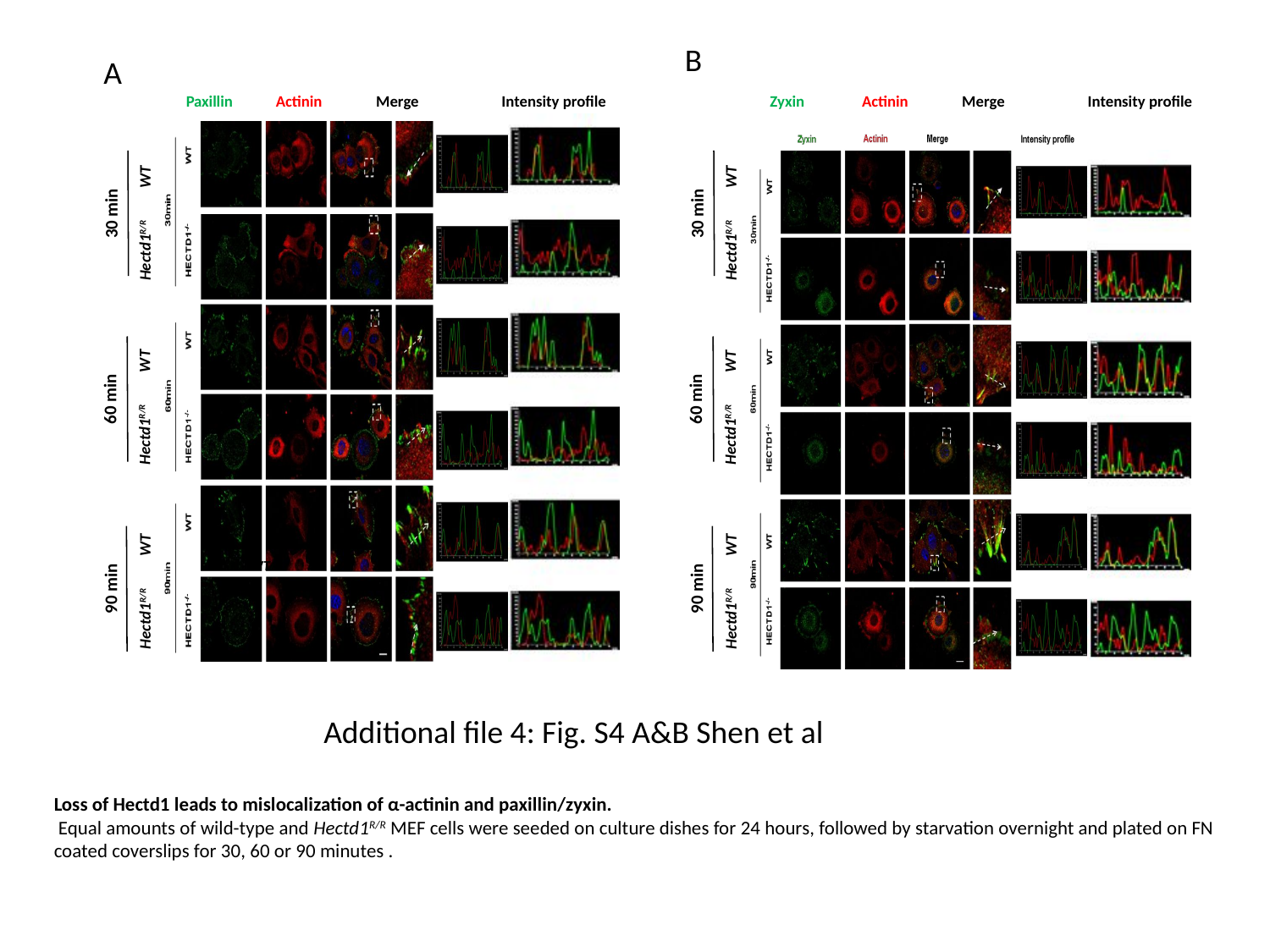

B
A
Paxillin Actinin Merge Intensity profile
Zyxin Actinin Merge Intensity profile
Hectd1R/R WT
30 min
Hectd1R/R WT
60 min
Hectd1R/R WT
90 min
Hectd1R/R WT
30 min
Hectd1R/R WT
60 min
WT
Hectd1R/R WT
90 min
Additional file 4: Fig. S4 A&B Shen et al
Loss of Hectd1 leads to mislocalization of α-actinin and paxillin/zyxin.
 Equal amounts of wild-type and Hectd1R/R MEF cells were seeded on culture dishes for 24 hours, followed by starvation overnight and plated on FN coated coverslips for 30, 60 or 90 minutes .
